# Current News

**Published:** 1902-07

**Authors:** 


### Current News.

### NATIONAL DENTAL ASSOCIATION.

The sixth annual session will be held in Niagara Falls, N. Y., July 28 to 31, 1902.

A good programme is being prepared and a large and profitable meeting is anticipated.

A rate of one fare and a third for the round trip, on the certificate plan, has been secured on all roads in the United States and part of Canada.

In purchasing ticket going, full fare must be paid and a railroad *certificate* taken; this when properly signed entitles holder to return for one-third fare.

Tickets may be bought going from July 22 to 29. The certificates for return journey may be used as late as August 4.

A. H. Peck,

*Recording Secretary.*

Chicago.

### NATIONAL ASSOCIATION OF DENTAL FACULTIES.

The nineteenth annual convention of the National Association of Dental Faculties will convene in the ball-room of the International Hotel, Niagara Falls, New York, July 24 next. The Executive Committee will meet at eleven A.M., July 23.

All colleges are respectfully referred to the rule requiring that their annual announcement be in the hands of the Executive Committee at this meeting.

H. B. Tileston,
*President.*

S. W. Foster,
*Secretary Executive Committee, N. A. D. F.*

---

### PENNSYLVANIA STATE DENTAL SOCIETY.

The Pennsylvania State Dental Society will hold its regular annual meeting at Bedford Springs Hotel, Bedford, Pa., July 8, 9, and 10, 1902.

R. H. Nones,
*Chairman Executive Committee.*

---

### ILLINOIS STATE DENTAL SOCIETY.

At the annual meeting of this Society, held at Springfield, May 13 to 15, 1902, the following officers were elected for the ensuing year:

President, A. H. Peck, Chicago; Vice-President, W. E. Holland, Jerseyville; Secretary, Hart J. Goslee, Chicago; Treasurer, C. N. Johnson, Chicago; Librarian, J. T. Cummins, Metropolis; Supervisor of Clinics, C. P. Pruyn, Chicago.

*Committee on Science and Literature.*—G. V. Black, Chicago.

*Committee on Art and Invention.*—L. S. Tenney, Chicago.

*Board of Examiners.*—C. B. Sawyer, Jacksonville.

*Committee on Ethics.*—E. A. Royce, Chicago; G. E. Warren, Pontiac; E. F. Hazell, Springfield.

*Executive Committee.*—Chas. J. Sowle, Rockford.

*Members of Executive Council.*—E. K. Blair, Waverly; D. M. Gallie, Chicago; O. M. Damude, Monmouth.

*Publication Committee.*—Hart J. Goslee, Chairman; D. M. Cattell, G. W. Dittmar, Chicago.

*Local Committee of Arrangements.*—F. M. McIntosh, J. B. Brown, G. D. Sitherwood, all of Bloomington, which city was selected as the next place of meeting.

HART J. GOSLEE,
*Secretary.*

---

### CHICAGO DENTAL SOCIETY.

FOLLOWING are the officers of the Chicago Dental Society elected at the annual meeing, April 1, 1902:

President, Elgin Mawhinney; First Vice-President, H. J. Goslee; Second Vice-President, F. B. Noyes; Secretary, Winthrop Girling; Corresponding Secretary, C. S. Bigelow; Treasurer, E. R. Carpenter; Librarian, H. W. Sale; Member of Board of Directors, Edmund Noyes.

*Board of Censors.*—W. V.-B. Ames, Chairman; C. N. Johnson, A. W. Harlan.

C. S. BIGELOW,
*Corresponding Secretary.*

---

### MISSOURI STATE DENTAL ASSOCIATION.

AT the last annual meeting of this Association, held at Jefferson City, May 21, 22, and 23, 1902, the following officers and committees were elected:

President, S. C. A. Rubey, Clinton; First Vice-President, J. H. Kennerly, St. Louis; Second Vice-President, F. W. Franklin, Kansas City; Corresponding Secretary, Otto J. Fruth, St. Louis; Recording Secretary, H. H. Sullivan, Kansas City; Treasurer, J. T. Fry, Moberly.

*Board of Censors.*—A. M. McGee, Louisiana; R. J. Winne, Bolivar; W. M. Bartlett, St. Louis.

*Committee on Ethics.*—A. J. Prosser, St. Louis; W. H. Renoe, Fulton; J. B. McBride, Springfield.

*Publication Committee.*—Wm. Conrad, St. Louis; W. G. Goodrich, Chillicothe.

*Committee on History.*—Burton Lee Thorpe, St. Louis.

*Committee on Inventions and New Appliances.*—Sam. T. Bassett, St. Louis.

*Committee on International Dental Congress during Louisiana Purchase Exposition.*—Wm. Conrad, F. F. Fletcher, M. C. Marshall, Hermann Prinz, Burton Lee Thorpe, W. M. Bartlett, Leo Gregory McKellops.

Next annual meeting will be held at Kansas City, Mo.

OTTO J. FRUTH,
*Corresponding Secretary.*

---

### MAINE DENTAL SOCIETY.

THE Maine Dental Society will hold its thirty-seventh annual meeting at Camden, Me., on Tuesday, Wednesday, and Thursday, July 15, 16, and 17, 1902.

H. A. KELLEY,
*Secretary.*

PORTLAND, ME.

---

### AMERICAN DENTAL SOCIETY OF EUROPE.

FOLLOWING is a synopsis of the programme for the annual meeting of the American Dental Society of Europe, to be held in Stockholm, August 12 to 15, 1902, inclusive:

TUESDAY, AUGUST 12.

9.30 A.M.—Business meeting (members only), Grand Hotel.
10.30 A.M.—Meeting open to visitors. Papers.
10.45 A.M.—President's Address.
1.00 P.M.—Adjournment for luncheon at various hotels.
2.30 P.M.—Meeting resumed.
9.00 P.M.—Reception by the President and Mrs. Royce. Music and dancing.

During the day drives and entertainments will be arranged for the ladies.

WEDNESDAY, AUGUST 13.

10.00 A.M.—Papers and discussions.

1.00 P.M.—Adjournment. Luncheon to visitors by members A.D.S.E.

2.30 P.M.—Meeting resumed.

7.30 P.M.—Dinner at one of the resorts of the season for ladies and members.

Excursion for the ladies to environs of Stockholm.

THURSDAY, AUGUST 14.

10.00 A.M.—Papers and discussions.

1.00 P.M.—Adjournment for luncheon.

2.30 P.M.—Meeting resumed.

8.00 P.M.—Annual banquet.

An all-day trip will be arranged for the ladies, returning in the evening.

FRIDAY, AUGUST 15.

9.30 A.M.—Clinics and demonstrations.

1.00 P.M.—Adjournment for luncheon.

2.30 P.M.—Business meeting.

Adjournment.

L. J. MITCHELL,
*Honorary Secretary.*

---

### VIRGINIA STATE DENTAL ASSOCIATION.

THE Virginia State Dental Association will meet at the Hygeia Hotel, Old Point Comfort, Va., August 5, 6, and 7, 1902. Visiting dentists will receive a cordial welcome.

J. HALL MOORE,
*Corresponding Secretary.*

